# Raid on the Uterus

**Published:** 1867-08

**Authors:** 


### Raid on the Uterus.

A distinguished surgeon in New York City, twenty-five years ago said, when Dupuytren's operation for relaxation of the *sphincter ani* was in vogue, every young man who came from Paris found every other individual's anus too large, and proceeded to pucker it up. The result was that New York anuses looked like gimlet-holes in a piece of pork. It seems to me that just such a raid is being made upon the uterus at this time. It is a harmless, unoffensive little organ, stowed away in a quiet place. Simply a muscular organ, having no function to perform save at certain periods of life, but furnishing a capital field for surgical operations, and is now-a-days subject to all sorts of barbarity from surgeons anxious for notoriety. Had Dame Nature foreseen this, she would have made it iron-clad. What with burning and cauterizing, cutting and slashing, and gouging, and spitting and skewering, and pessarying, the old-fashioned womb will cease to exist, except in history. The *Transactions* of the National Medical Association for 1864 has figured one hundred and twenty-three different kinds of pessaries, embracing every variety, from a simple plug to a patent threshing machine, which can only be worn with the largest hoops. They look like the drawings of turbine water-wheels, or a leaf from a work on entomology. Pessaries, I suppose, are some times useful, but there are more than there is any necessity for. I do think that this filling the vagina with such traps, making a Chinese toy-shop of it, is outrageous. Hippocrates said that he would never recommend a pessary to procure abortion—nay, he swore he never would. Were he alive now he would never recommend one at all. If there were fewer abortions there would be fewer pessaries, and if there were fewer pessaries there would be fewer abortions. Our grandmothers never knew they had wombs only as they were reminded of it by the struggles of a healthy fœtus; which, by the by, they always held on to. Now-a-days, even our young women must have their wombs shored up, and if a baby accidentally gets in by the side of the machinery, and finds a lodgment in the uterus, it may, perchance, have a knitting-needle stuck in its eyes before it has any. It is the easiest thing in the world to introduce a speculum and pretend to discover ulceration of the os, and subject a patient to this revolting manipulation once or twice a week, when there is, in fact, nothing the matter. By some practitioners, all diseases which occur in the female are attributed to the uterus. In this class are especially to be included all such as make of the abnormal conditions of the uterus a specialty.—*Extract from the Address of Dr. W. D. Buck, President of the New Hampshire State Medical Society for* 1866.—*New York Medtcal Journal.*

